# Combinatorial design of calcium meta phosphate poly(vinyl alcohol) bone-like biocomposites

**DOI:** 10.1007/s10856-018-6133-6

**Published:** 2018-07-30

**Authors:** Shathani Nkhwa, Lilis Iskandar, Neelam Gurav, Sanjukta Deb

**Affiliations:** Tissue Engineering & Biophotonics, King’s College London, Dental Institute, Floor 17, Tower Wing, Guy’s Hospital, London Bridge, London, SE1 9RT UK

## Abstract

The incidence of degenerative diseases and the ageing population have added to the growing demand for bone grafts. Although autologous bone continues to be the gold standard, limited yield and potential morbidity of the donor site pose considerable challenges. Currently, clinically used synthetic grafts based on calcium phosphates are mechanically brittle and not compliant hence composite scaffolds are expected to be provide viable solutions. In this study we report composites of calcium meta phosphate-poly (vinyl alcohol) with tunable mechanical properties, low swelling and excellent biocompatibility. The elastomeric nature of the composites resist brittle fracture and the scaffolds can be easily shaped to the bone defect by the surgeon. Testing on bone plug shaped specimens of the scaffolds, exhibited superior mechanical properties compared to currently commercially available bone plugs with additional advantages being the ability to increase porosity without compromising properties in compression and degree of swelling, which make these composites promising synthetic alternatives for bone grafts and bone tissue engineering.

## Introduction

Strategies in designing bone substitutes include creating a 3D matrix environment that mimics the extracellular matrix, enabling osteogenesis by allowing for the attachment of osteoprogenitor cells to proliferate and differentiate into osteoblasts whilst simultaneously providing the requisite mechanical integrity, which is integral in bone regeneration. Although autografts continue to outperform synthetic substitutes, the strategy of mimicking the non-stoichiometric and substituted calcium phosphates of the bone mineral in designing synthetic substitutes have shown promise but with its own limitations [1]. There is an array of synthetic calcium phosphate based biomaterials ranging from calcium hydroxyapatite (HA), Ca_10_(PO_4_)_6_(OH)_2_; alpha/beta-tricalcium phosphate (α- or β-TCP), Ca_3_(PO_4_)_2_; biphasic calcium phosphates (BCPs); mixtures of HA and β-TCP; and unsintered apatites or calcium-deficient apatites (CDA), however β-tricalcium phosphate (β-TCP) and hydroxyapatite (HAp) are most frequently used due to their reported biocompatibility and osteoconductivity [2–6]. The solubility of phosphate biomaterials is known to differ, hydroxyapatite being the most stable, however the variation in crystallinity, porosity, architecture and topography contribute to the biological performance despite the extensive evidence of the osteogenic and osseointegrative properties of different calcium phosphates. Calcium phosphates are a material of choice for bone substitution and forming composites with polymers lead to a multiphase system consisting of a matrix reinforcement of various shapes and sizes, with resultant properties superior to those of the individual components. The CaP fillers used to reinforce polymer matrices play a special role in the desired properties of the composite biomaterial, where an increase in surface area of the inorganic filler significantly enhances the mechanical properties of the material and influences the degradation rates and bioactivity of the biomaterial. However, composites tend to have non-uniform dispersion of fillers within the polymer matrix, as well as insufficient interaction at the phase interface and agglomerates in nano-sized reinforcements, all of which are major drawbacks in composite formulations of biomaterials. Although there is a large number of combinatory design strategies using calcium phosphates reported in literature [7], it is becoming evident that future design of scaffolds for bone tissue regeneration is likely to be more successful through localised delivery of growth factors, cytokines, anti-infectives both in acellular or cellular scaffolds for bone regeneration [8].

In this paper we report designing of hydrogel composites with calcium meta phosphate as the resorbable mineral phase with poly (vinyl alcohol) (PVA) forming the matrix. Since the function of a bone substitute is transient, a meta stable phase, namely a calcium meta phosphate (CMP) scaffold with an interconnected porous architecture in our previous study [9] was shown to form new bone in rabbit maxillary critical sized bone defects within eight weeks with little evidence of any remaining scaffold. An addition of osteogenic protein-1 (OP-1) within these scaffolds enhanced the rate of bone formation, nevertheless the CMP on its own exhibited excellent bone growth and full coverage of the defect. However, one of the main drawbacks of this scaffold is the fragility and brittleness, which necessitates pre-fabrication to fit the bone defect and does not allow further manipulation in theatre by the surgeon.

PVA is a biocompatible polymer that has been widely used in biomedical engineering and pharmaceutical technology applications [10]. It has a simple structure, which can be easily tailored depending on the application, and also has the ability to form crosslinked structures without the incorporation of toxic additives [10]. A recent review [11] details the formulation of calcium phosphate-PVA composites that eloquently discusses the two main pathways of PVA-HA composites either via production of inorganic/organic composite hybrids through precipitation of the inorganic phase in PVA or direct composite formulation through addition of small amounts of hydroxyapatite in PVA and subjecting them to freeze-thaw cycles.

The PVA-CMP composites in this study were designed as bone plugs suited for use in maxillofacial and other bone defects. The composites were formulated to be enriched with the resorbable calcium metaphosphate and enable gelation without use of harsh chemicals whilst imbibing porosity through the elimination of the ice crystals formed via freeze-thawing of the matrix phase. The presence of the hydrogel matrix would additionally facilitate incorporation of orthobiologics and drugs or growth factors. The inclusion of PVA in the network was postulated to be able to inherently reduce the brittleness of the scaffold and resorption rate of the CMP filler phase in the composite. Hence the concentration of PVA was varied to formulate the composites to study the influence on the properties whilst a maximum permissible amount of the filler was used and kept constant for all the formulations based on the feasibility of obtaining homogenous dispersion of the filler in the viscous solution of PVA. The composites obtained were characterised to determine the effect of incorporation of CMP filler particles as well as the effect of variation of PVA concentration on the properties of the composites. Furthermore, a study on including particulate gelatin as a porogen in the PVA-CMP composite to enhance macroporosity is also reported.

## Materials and methods

Poly(vinyl alcohol) (PVA), 145,000 mol. wt. hydrolysis ≥98% (Merck, Schuchardt OHG). CMP (Calcium metaphosphate) Ca(PO_3_)_2_ (prepared in house, method by Deb et al). MCPM (Monocalcium phosphate monohydrate) Ca(H_2_PO_4_)2.H_2_O (M = 252.07) Scharlab S.L

### Preparation and fabrication of PVA-CMP composites

**Fig. 1:**
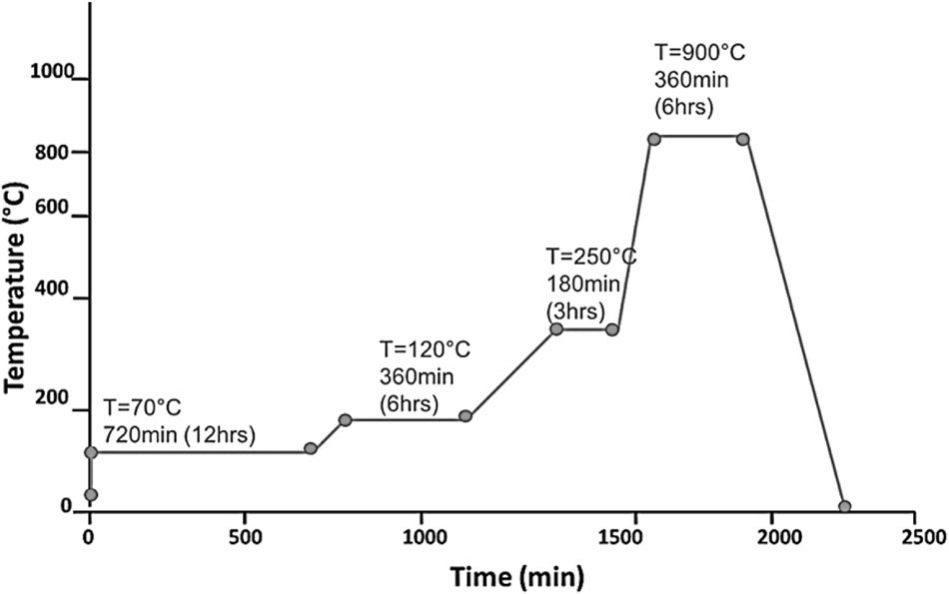
Temperature programme for sintering MCPM to CMP, time required by thermal process ~36 h (including cooling step)

Filler phase: The filler particles were prepared by subjecting monocalcium phosphate monohydrate (MCPM) powders compressed into pellets to a sintering regime shown in Fig. 1. The resultant mass was cooled and milled to a fine powder and the phase changes of MCPM to CMP were analysed by FTIR and XRD. The particle size was determined using a CILAS 1180 laser diffraction particle analyser (Cilas, Orleans; France) operating at an 830 nm central wavelength and 7 mW energy power.

Matrix phase: Aqueous solutions of PVA 10, 20 and 30% w/v were prepared by dissolution of the appropriate amount of PVA granules in distilled water and magnetically stirred at 121 °C until all the PVA granules dissolved to form a homogenous solution.

Composite: The aqueous PVA solutions were mixed with CMP powder using ratio 1:1.5 (40PVA:60CMP percent by weight) so as to obtain a paste like consistency. The amount of CMP was kept constant based on a pilot study that was designed to incorporate the highest amount of CMP yielding a mouldable paste. This mixture was subjected to centrifugation at 3400 rpm for 15 min after which any excess PVA was decanted. The paste was then subjected to freeze drying for 24 h, and placed in desiccator immediately. All composites were prepared by 1 cycle of freeze drying and thawing (1FT).

Composites with porogen: A 10% PVA solution was used to blend with 60% by weight of CMP and 10% by weight of gelatin added with respect to PVA. The CMP was first homogenously mixed into the PVA solution and subsequently gelatin granules were added to the paste and dispersed. The pastes were moulded in cylindrical tubes and subjected to 1 cycle of freeze-thawing as described for the PVA-CMP composite. The porogen was allowed to swell in distilled water and removed by maintaining the temperature at 37–40 °C.

### Attenuated total reflectance fourier transform infrared spectroscopy (ATR–FTIR)

ATR/FTIR spectra of the composites were recorded on a Perkin Elmer Spectrum One spectrometer. The gels were placed in a desiccator for 24 h prior to testing, and all spectra were obtained in the wavelength range of 4000–650 cm^−1^ with 4 cm^−1^ resolution.

### Differential scanning calorimetry (DSC) analysis

A Perkin Elmer Jade series differential scanning calorimeter was used to determine the thermal properties and Perkin Elmer Jade series software to process raw data. 10–20 mg samples were carefully placed and sealed in aluminium pans (Perkin Elmer). The scans were carried out with reference pan calibrated using Indium^49^ under a Nitrogen^7^ atmosphere. Two cycles of heating and cooling were carried out, starting from 0 to 250 °C followed by a cooling cycle to 10 °C at a rate of 10 °C per min. The temperature of the second heating cycle was raised to 250 °C and the same cooling cycle followed. The glass transition temperature (T_**g**_°C) and melting temperature (T_**m**_°C) were calculated using the Pyris Jade DSC (Perkin Elmer) software.

### Equilibrium water content (EWC)

Hydrogels were immersed in deionised water at 37 °C and mass monitored using conventional gravimetric methods until equilibrium was reached. The EWC and degree of swelling of the xerogels were determined using the following equation:

$$EWC = \frac{{Ws - Wd_0}}{{Ws}} \times 100{\mathrm{\% }}$$

The degree of swelling in water was determined by dividing the equilibrium weight in distilled water at room temperature while the dry gel mass was determined by drying the gel samples to constant weight under vacuum at 30 °C.

$$Swelling\,Ratio\,\left( {SR} \right) = \frac{{Ws}}{{Wd_0}}$$

The gel fraction was determined using the dried gel mass weight to the initial mass weight of the polymer.

$$Gel\,fraction = \frac{{Wd_1}}{{Wd_0}} \times 100{\mathrm{\% }}$$

*Ws:* equilibrium (hydrated) weight, *Wd*_*0:*_ initial dry weight and *Wd*_*1*_: final dry weight

### Compression and diametral compression tests

A universal testing machine (Instron 5569 A) with a load cell of 50kN and a crosshead rate of 0.5 mm/min was used to determine the compression carried out on cylindrical specimens with dimensions (ϕ = 4 mm, h = 6 mm). Diametral compression was carried out on specimens with dimension d = 12 mm and t = 6 mm. The load to failure (P) obtained, was used to calculate strength using the formula σ_T_ = 2 P/πDt, where P is the indirect tensile load, d and t are the diameter and thickness of the specimens respectively.

### Scanning electron microscopy (SEM) and EDAX

Scanning electron microscopy and EDAX was carried out on selected samples placed on aluminium stubs using conductive blue then coated in a thin layer of gold for quanta field emission scanning electron microscope (Quanta 200 F microscope (FEI).

### Statistical analysis

Statistical analysis was performed where appropriate using independent t-tests, one-way ANOVA and post-hoc Bonferroni or Tukey’s tests with level of significance set at *p* < 0.05 for all calculations (*p < 0.05; ** *p* ≤ 0.01; *** *p* ≤ 0.001; **** *p* ≤ 0.0001). All analysis was done in GraphPad Prism 7.03.

### Cytotoxicity evaluation of hydrogels

All test hydrogel composites were sterilised by gamma irradiation and human osteoblast-like cells (HOB) were used. Cytotoxicity was determined using an elution study (MTT assay) at 24, 48 and 72 h. The biomaterials were placed in DMEM cell culture media and placed on a roller for 24, 48 and 72 h. Cells were cultured in the elution media at 37 °C in a CO_2_ incubator. Surviving HOB cells were quantified using MTT assay, the positive control group 10% alcohol in media and negative control cells in media were adopted.

Cell adhesion studies were performed by culturing the HOB cells in the presence of the biomaterials for 1, 7 and 14 days, in cell culture medium before viability was analysed using a Live/dead viability/cytotoxicity kit (L-3224) from Invitrogen. Cells were incubated with 1 µM of calcein AM and 2 µM of ethidium homodimer in PBS and placed in CO_2_ incubator for 20 mins. Calcein stains the live cells green due to intracellular esterase activity, and ethidium stains the cells red as it enters cells with damaged membranes and becomes fluorescent upon binding to nucleic acids in the dead cell. The cells were imaged with a fluorescence microscope (Olympus IX51).

Cell morphology using SEM on hydrogels was carried out at time points 7 and 14 days, each sample was fixed with 1.5% glutaraldehyde buffered in 0.1 sodium cacodylate. The cells were stained in 1% osmium tetroxide and 1% tannic acid, and then dehydrated through a series of alcohol concentrations (20, 30, 40, 50, 60, 70, 90, 96 and 100%). The final air-drying was done in hexamethyl disilazane (HMDS). The samples were gold-palladium sputter coated for 2 min and viewed using a JOEL scanning electron microscope (SEM).

## Results

### X-ray diffraction (XRD)

**Fig. 2:**
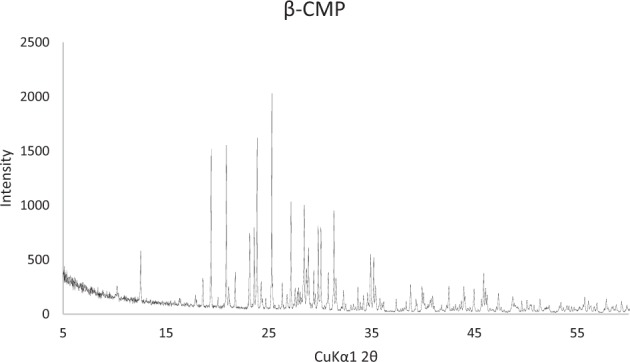
X ray diffraction pattern of the β meta calcium phosphate

The XRD spectrum of the filler obtained after sintering mono calcium phosphate monohydrate is shown in Fig. 2. The peaks were observed at 26°, 29° and 32° 2θ angles, which indicated the main phase as β-calcium metaphosphate. Other broader low intensity peaks, characteristic of calcium phosphates were observed at 40°, 47° and 50°.

### Attenuated Fourier transform infrared (AT-FTIR) spectroscopy

The FTIR spectra of CMP in Fig. 4, showed characteristic absorption peaks arising due to phosphate groups at 1061, 1115 and 1241 cm^−1^. The shoulder 790 cm^−1^ can be assigned to the covalent bond between non-bridging oxygen and calcium ions (P-O-Ca) stretching vibration.

The particle size analysis of the CMP powders exhibited a normal distribution with a mean particle size of 49.9 µm. The particle size distribution percentiles of CMP powder at 10, 50 and 90 % were found to be 6.2, 24.1 and 49.9 µm respectively.

**Fig. 3:**
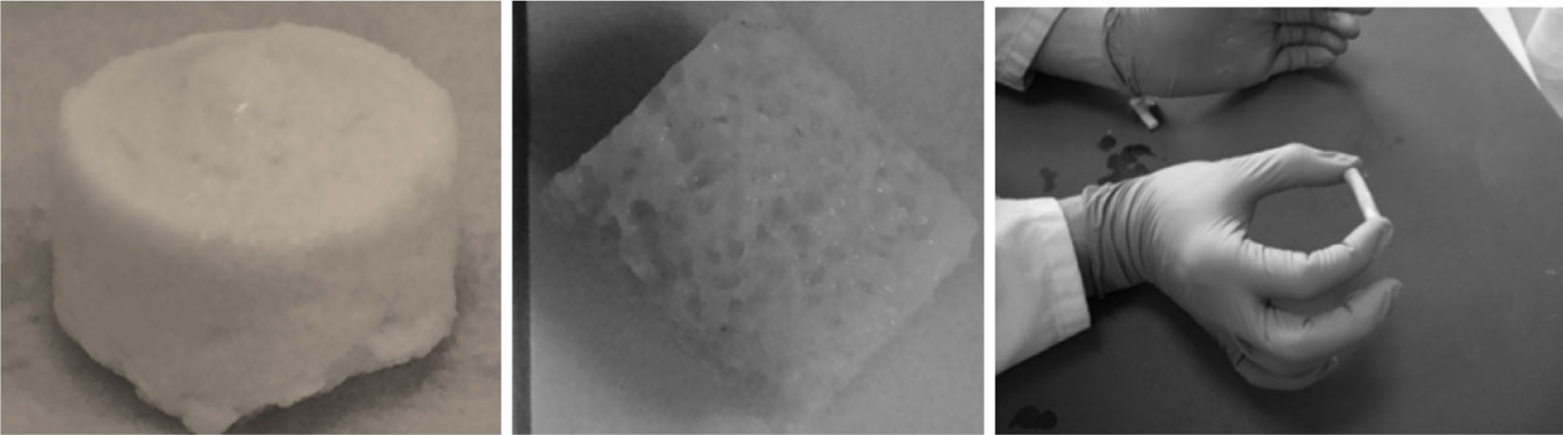
Images illustrating the PVA-CMP composites

**Fig. 4:**
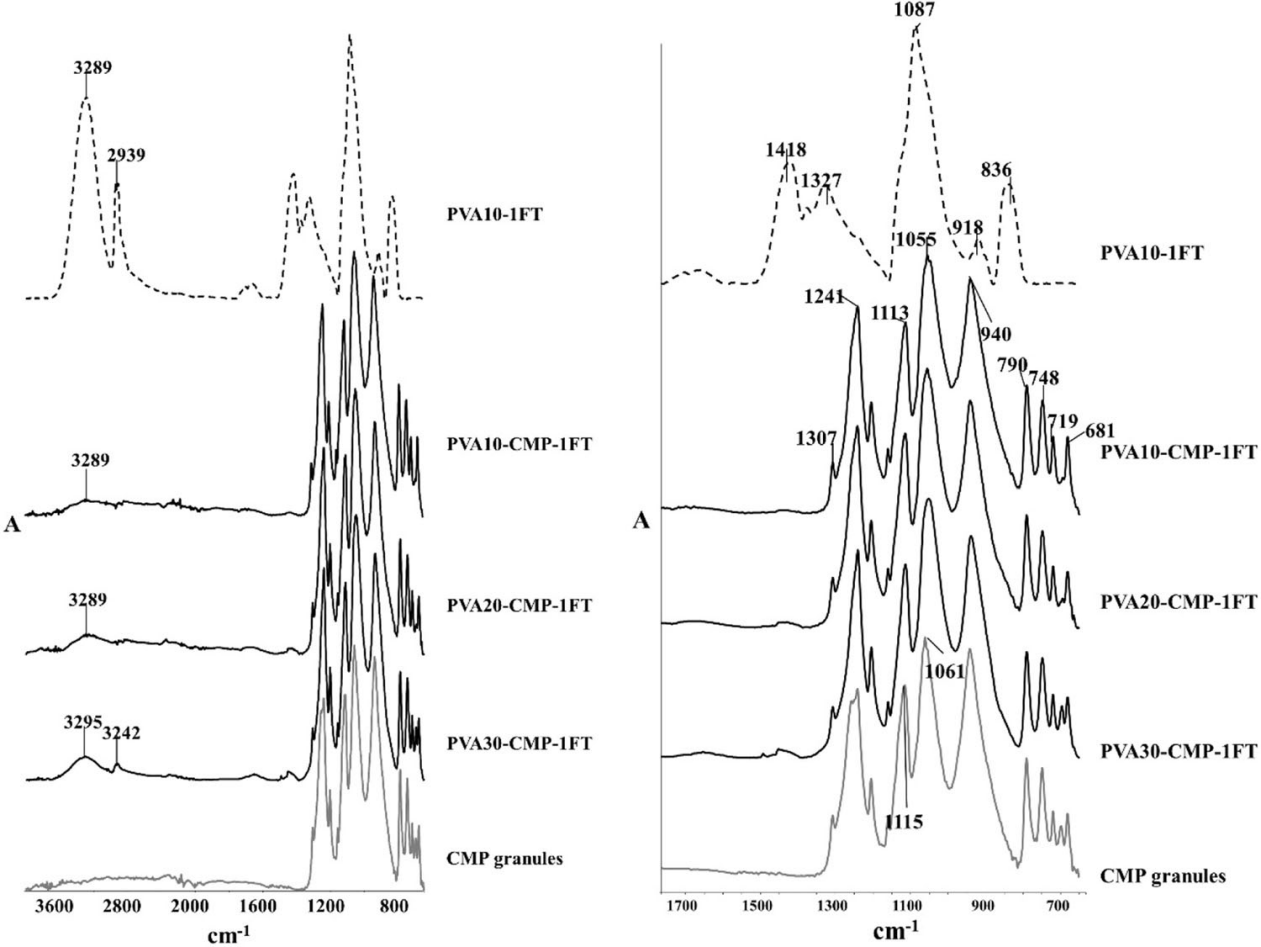
FTIR spectra of CMP granules, PVA10-1FT hydrogel and PVA-CMP composites fabricated by crosslinking using a single freeze thaw cycle and the expanded fingerprint region

**Fig. 5:**
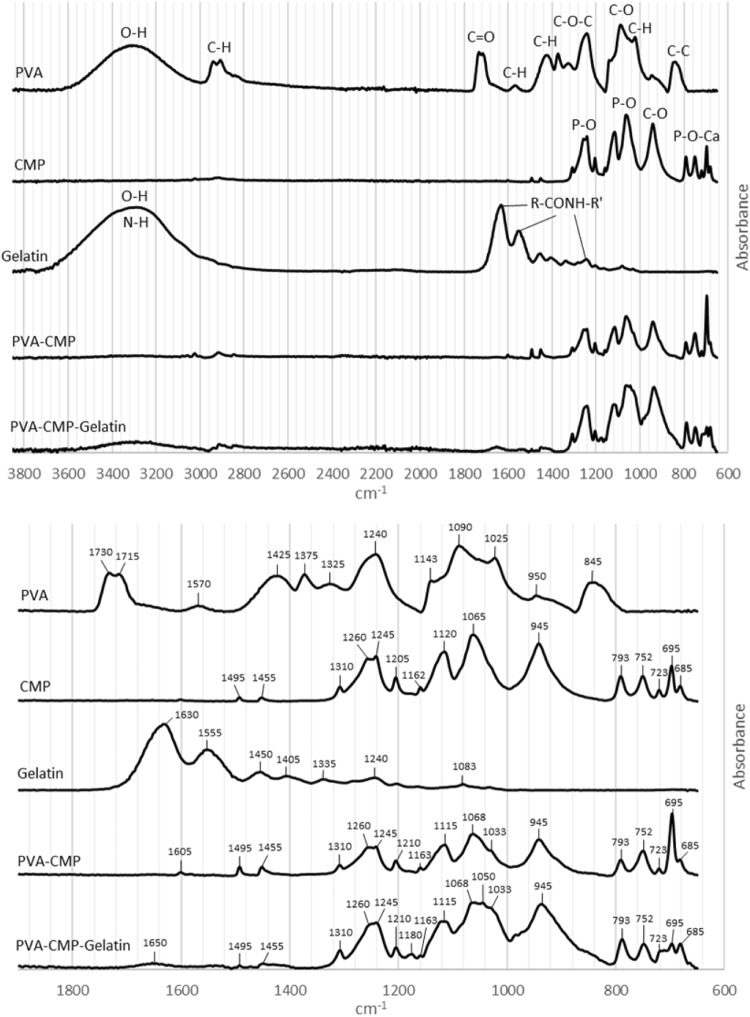
A comparison of the FTIR spectra of the components PVA, CMP, Gelatin and the composites without and with gelatin as porogen

PVA: CMP composites: Rigid sponge like composites (Fig. 3) were obtained after fabrication using the methodology described and the FTIR spectra of the hydrogel composites without and with porogen are shown in Figs. 4 and 5 respectively. The main characteristic peaks associated with both PVA and calcium metaphosphate was evident in the spectra of the composites. The stretching frequency of CH_2_ groups of the PVA were observed through peaks at 2920 cm^−1^ (symmetric), 2850 cm^−1^, 1420–1492 cm^−1^(bending/scissoring), 1280–1315 cm^−1^(wagging) and 1167–1229 cm^−1^ (twisting) [5]. The peaks at 1418 cm^−1^ arises due to the -CH_2_ bending with deformation bands of C-CH_3_ appearing at 1327 cm^−1^. The -C-OH(~1088 cm^−1^) is related to the symmetric C-C stretching mode or C-O stretching where an intramolecular hydrogen bond is formed between two neighbouring hydoxyl groups on the same side of the plane of the carbon chain backbone. The strong absorption peak arising due to hydroxyl groups (3289 cm^−1^) and C-H stretching of alkyl groups (2939 cm^−1^) of PVA (observed on the PVA-1FT spectra; Fig. 4) were not as prominent and much weaker in the PVA-CMP composites due to the high concentration of CMP in the composite that yielded significantly high intensity bands. The increase in the intensity of the peak due to hydroxyl groups increased in the PVA30-CMP at 3295 cm^−1^ as expected due to the increasing concentration of PVA in the composite formulation. A slight shift in the phosphate peaks of PVA-CMP composites observed at 1113 and 1055 cm^−1^ is attributed to specifically the C-OH bending vibration at 1087 cm^−1^, which may be evidence of intermolecular interaction between PVA and CMP. However prominent peaks attributed to CMP were observed in the composites at 681 cm^−1^ and 1168 cm^−1^ correlating to the P-O-P and PO_4_^3−^ vibrations respectively, which are in agreement with other studies [12]. The peaks at 1000 cm^−1^ correlates to the out of plane bending of C-H groups in PVA. A low intensity peak at 1630 cm^−1^ and the broad peak at 3212 cm^−1^ can be attributed to the amide stretching and the OH and -NH stretching indicates that gelatin is present to a certain extent in the composite (Fig. 5), which may be due to the weak interaction between PVA and gelatin.

### Glass transition temperature

The glass transition temperature of the PVA-CMP composites showed no statistically significant difference between the different concentrations of PVA (Table 1) used to fabricate the composites.

**Table 1:** Glass transition temperatures (Tg°C) of the PVA 10-CMP, PVA20 and PVA30-CMP composites fabricated by one cycle of freeze-thawing

Hydrogel composites fabricated by 1FT cycle	glass transition T_g_ (°C)	T_m_ (°C) (mean ± SD) (°C)
PVA10-CMP	106.4 ± 0.6	214.0 ± 1.4
PVA20-CMP	105.3 ± 0.4	
PVA30-CMP	105.4 ± 0.5	
PVA-CMP with porogen	118.1 ± 6.8	227.9 ± 0.2

### Water uptake

**Fig. 6:**
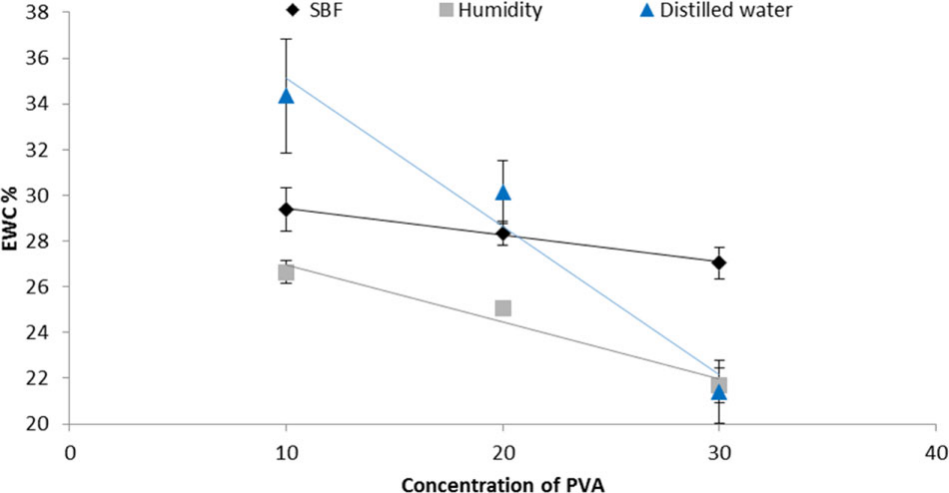
EWC of the PVA-CMP-1FT composites fabricated with PVA of concentrations 10, 20 and 30%. The scatter plot presents EWC of composites in SBF, 100% humidity and distilled water (*n* = 3)

**Fig. 7:**
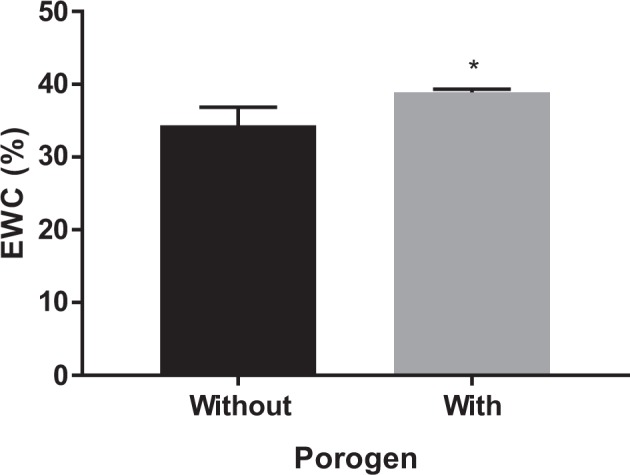
A comparison of the EWC of PVA10-CMP composites without and with gelatin as porogen

The equilibrium water uptake, swelling ratio and gel fraction of the composites with the three concentrations of PVA showed a gradual decrease with increasing concentration of PVA (Table 2). The EWC of the composites were also determined in three different biologically relevant environments being distilled water (DW), simulated body fluid (SBF) and 100% humidity. The results are summarized in Fig. 6 that indicated the highest EWC (*p* ≤ 0.032) were obtained on immersion in SBF and the 10% PVA composites exhibited higher EWC in all three media as compared to PVA20-CMP and PVA30-CMP respectively for SBF, DW and humidity, with the exception of PVA20-CMP in SBF. The EWC of the PVA10-CMP composite with porogen showed slightly higher values (*p < 0.05) than the corresponding composite with no porogen (Fig. 7).

**Table 2:** Equilibrium water content, swelling ratio and gel fraction of PVA-CMP composites prepared by one cycle of freeze-thawing. Tests carried out in distilled water (*n* = 3)

Hydrogel network obtained after 1-FT	EWC (%) distilled water	Swelling ratio (SR)	Gel fraction, GF (%)
PVA10-CMP	34.3 ± 2.5	1.5 ± 0.10	97.7 ± 0.2
PVA20-CMP	30.2 ± 1.4	1.4 ± 0.03	96.9 ± 0.9
PVA30-CMP	21.4 ± 1.4	1.3 ± 0.02	90.9 ± 4.1
PVA-10 CMP with porogen	38.9 ± 0.4	1.6 ± 0.01	97.1 ± 0.2

### Compressive and diametral properties

**Fig. 8:**
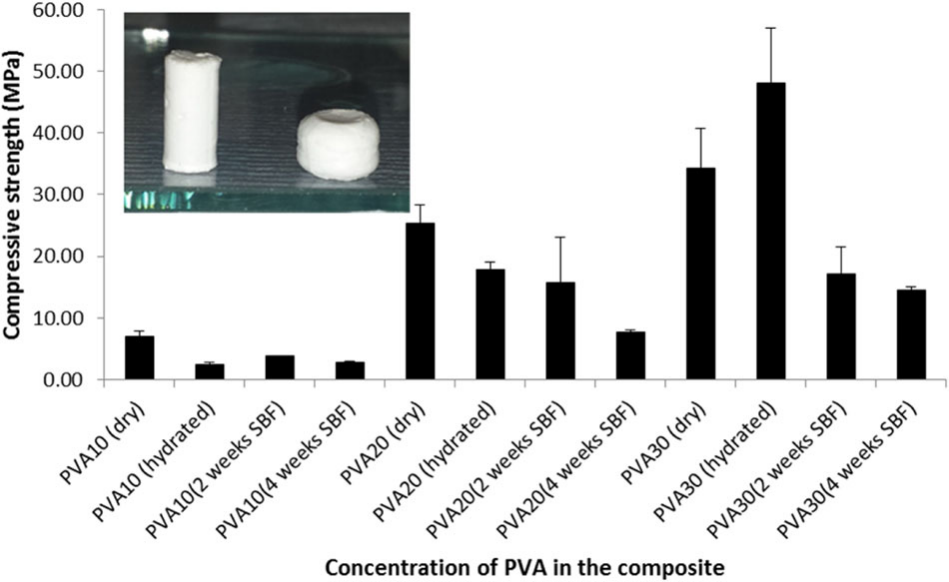
Compressive strength of PVA-CMP composites fabricated with PVA of concentrations 10, 20 and 30% and crosslinked via one cycle of freeze-thawing. Tests were carried out for both dry and hydrated specimens (*n* = 6), as well as after 2 and 4 weeks immersion in SBF (*n* = 4). As the materials are not brittle it should be noted that the values are comparative within the group. The inset is an image of PVA-CMP composite before compression (left) and after compression (right)

**Fig. 9:**
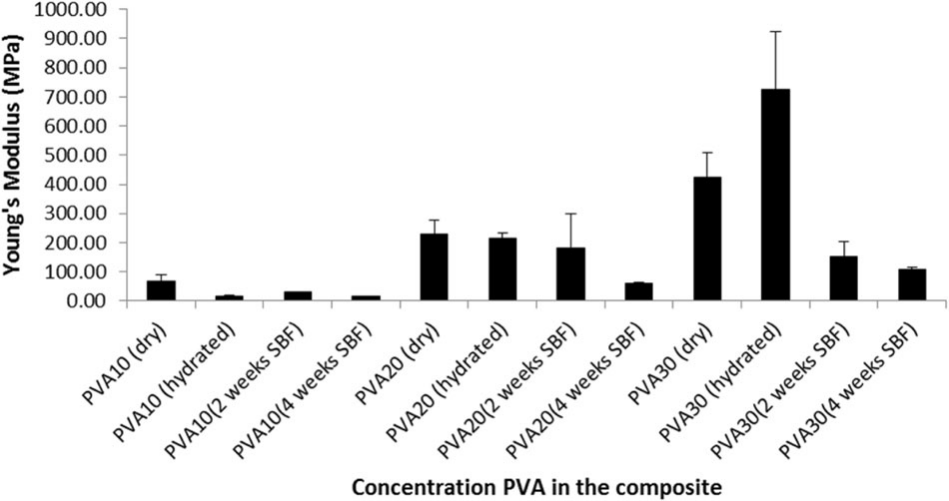
Young’s Modulus of PVA-CMP composites fabricated with PVA of concentrations 10, 20 and 30% and crosslinked via one cycle of freeze thawing. Tests were carried out for dry and fully hydrated specimens (*n* = 6), as well as after 2 and 4 weeks immersion time in SBF (*n* = 4). As the materials are not brittle it should be noted that the values are comparative within the group

The hydrated composite specimens when subjected to compression did not undergo brittle fracture but compaction occurred as the pores collapsed and the moisture released as demonstrated in Fig. 8. The compressive strength of the dry and hydrated (SBF) PVA-CMP composites were found to increase with increasing concentration of PVA as shown in Fig. 8. The composites in the dry state exhibited significantly higher (*P* ≤ 0.006) compressive strength than the hydrated composites PVA10 and 20-CMP (7.0 and 25.4 MPa respectively) with the exception of PVA30-CMP. The hydrated PVA30-CMP composite was found to have a significantly higher (*P* ≤ 0.005) compressive strength of 48.0 MPa than all the groups tested. This was due to the fact that the higher concentration of PVA results in a dense elastic network, which provides enhanced reinforcement in combination with CMP filler. This network when hydrated results in a higher resistance to larger loads of compression as compared to PVA10 and 20 concentration which are less dense. The compressive stiffness of the composites (Fig. 9) was also found to increase with increasing concentration of PVA and composites tested under dry conditions were found to have higher modulus values than those tested under hydrated conditions with exception of PVA30-CMP. The porous composite PVA10-CMP with gelatin as a porogen exhibited as shown in Table 3 lower compressive strength in the dry state as expected, however the compressibility increased in the hydrated state, which further confirms the presence of residual gelatin in the matrix that forms a network.

**Table 3:** The mean compressive strength and modulus of the dry and hydrated non-porous and porous composites of PVA10-CMP

Specimens		Compressive strength (MPa) (mean ± SD)	Compressive modulus (MPa) (mean ± SD)
PVA10-CMP	Dry*	7.0 ± 0.8	68.3 ± 19.8
	Hydrated**	2.5 ± 0.2	16.0 ± 2.5
PVA10-CMP with porogen	Dry	4.6 ± 1.2	143.8 ± 45.4
	Hydrated	9.2 ± 0.1	165.0 ± 7.7

*Testing was conducted to failure for dry specimens

**Maximum compressibility for the hydrated specimens

**Fig. 10:**
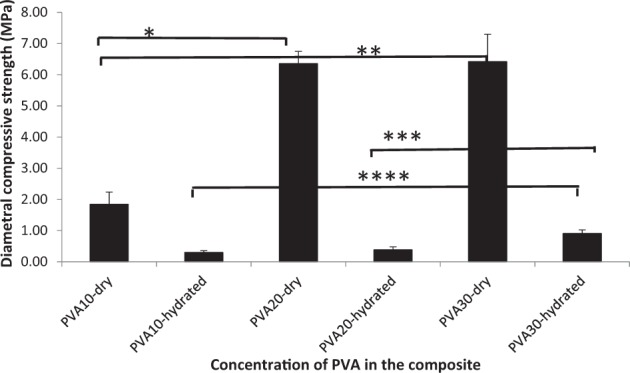
Diametral compressive strength of PVA-CMP composites fabricated with PVA of concentrations 10, 20 and 30% and crosslinked via one cycle of freeze thawing. Tests were carried out under dry and hydrated to equilibrium conditions. (*n* = 6) (*, **, *** and **** *P* < 0.001); asterisk linked with lines indicate significant difference in values between the groups. As the materials are not brittle it should be noted that the values are comparative within the group

**Fig. 11:**
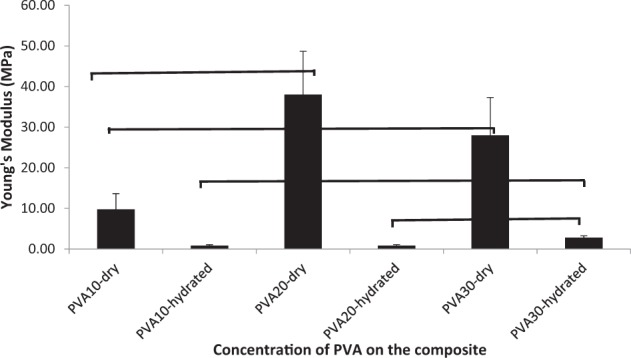
Diametral stiffness of PVA-CMP composites fabricated with PVA of concentrations 10, 20 and 30% and crosslinked via one cycle of freeze thawing. Tests were carried out under dry and hydrated to equilibrium conditions. (*n* = 6), (*, ***, **** *P* < 0.001 and ** *P* = 0.004); asterisk linked with lines indicate significant difference in values between the groups

Diametral compression tests (Figs. 10 and 11) were carried out on the composites, using cylindrical specimens similar to bone plugs in dental sockets. This is an indirect tensile test via induction of the local tensile stress in the transverse direction of the applied compressive stress, estimating the strength of materials as they undergo elastic deformation. Results indicated that diametral compressive strength (*P* < 0.001) and elastic modulus (*P* ≤ 0.004) under dry conditions were found to be highest with PVA20 (6.3 and 38.0 MPa) and 30-CMP (6.4 and 28.0 MPa) respectively. Under hydrated conditions strength and stiffness increased with increasing concentration of PVA, where PVA30-CMP had significantly (*P* ≤ 0.001) the highest strength and stiffness (0.9 and 2.8 MPa) compared to PVA10-CMP (0.3 and 0.8 MPa) and PVA20-CMP (0.4 and 0.9 MPa) respectively.

### Scanning electron microscopy

**Fig. 12:**
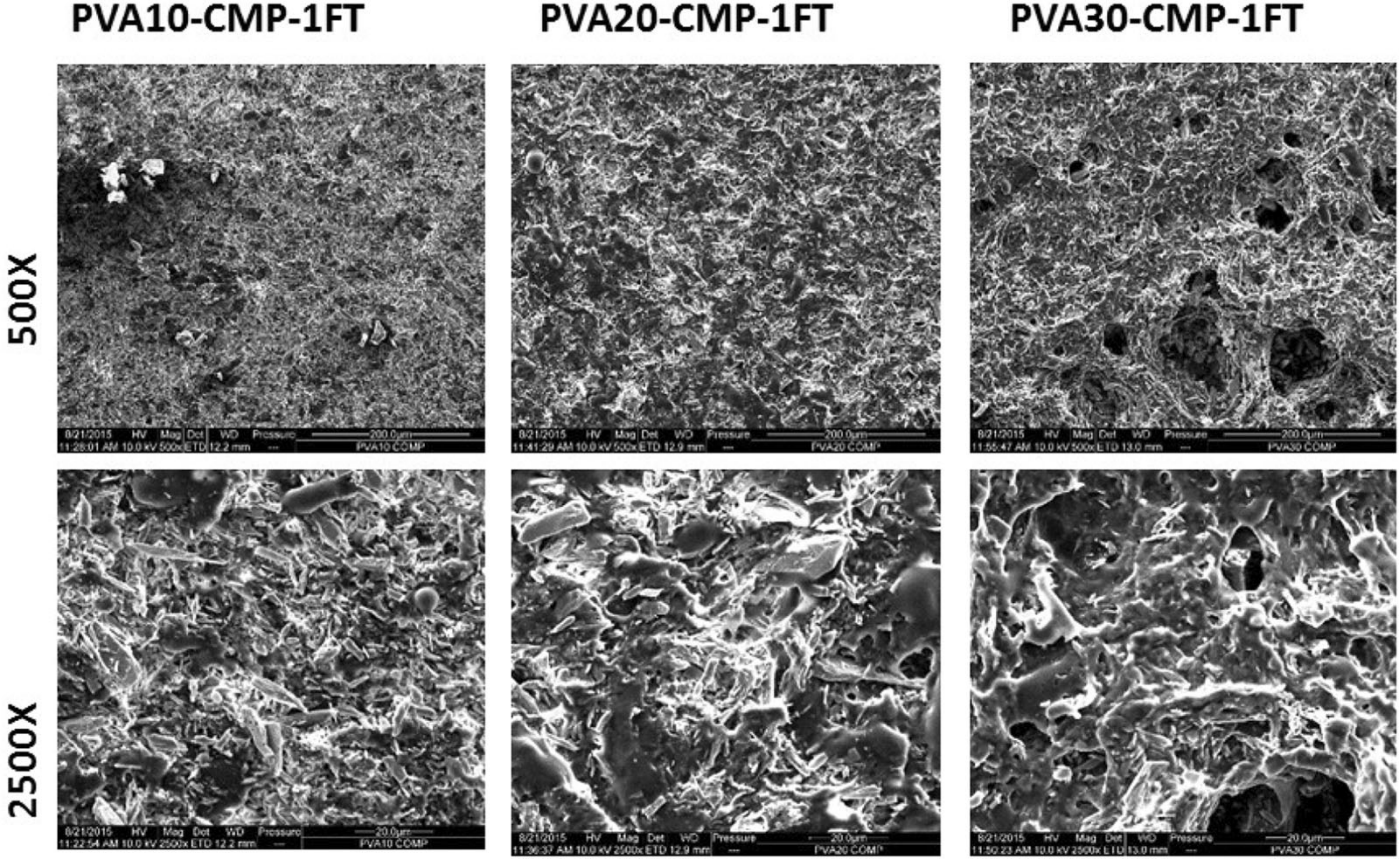
SEM micrographs showing the morphology of the freeze-thawed PVA-CMP composites starting with PVA10, 20 then 30 from top to bottom respectively, at magnifications 100×, 500× and 2500× from left to right respectively

**Fig. 13:**
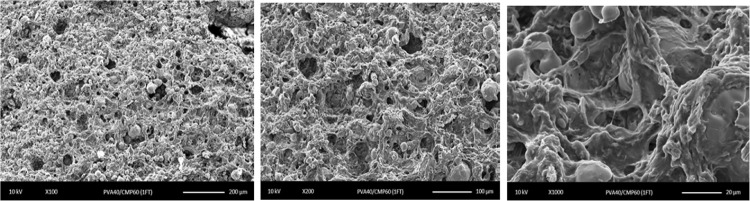
SEM micrographs showing the morphology of the freeze-thawed PVA10-CMP (gelatin as porogen) composites at 100×, 200× and 1000× from left to right respectively

The scanning electron micrographs of the PVA-CMP composites showed a network with a good distribution of the mineral phase in the PVA network. Spindle like crystals of CMP were visible along with micro-porosity in the matrix and the dense polymer phase was observed to increase with increasing PVA concentration as shown in Fig. 12. Figure 13 shows the PVA10-CMP composite at different magnification with evidence of macroporosity without the distinct appearance of the spindle like CMP crystals.

### Cytocompatibility

**Fig. 14:**
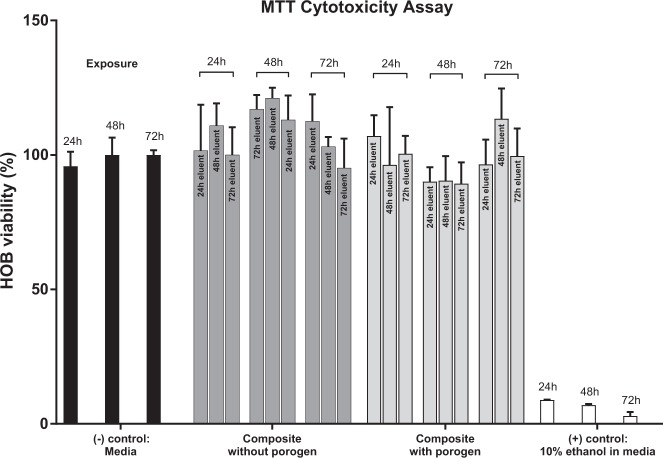
The viability of HOB cells in the eluents at 24, 48 and 72 h and exposed to the assay at 24, 48 and 72 h for the PVA10-CMP composites with and without gelatin as porogen

MTT assay is based on the reductive capacity of living cells to metabolize the tetrazolium salt, 3-(4,5-dimeththizaol-2yl)2,5-diphenyl tetrazolium bromide to a blue formazin product. The interaction of the eluents of the PVA10-CMP composite obtained at 24, 48 and 72 h were exposed to the MTT assay for 24, 48 and 72 h and there was no evidence of toxicity to the HOB cells. Similar results were obtained for the PVA 20-CMP and PVA30-CMP composites (results not shown). One-way ANOVA showed significant different among the specimens with slightly different significance level: *p* = 0.0004 (***) for 24 h exposure, *p* < 0.0001 (****) for 48 h exposure, and *p* = 0.002 (**) for 72 h exposure. Multiple comparisons (post hoc Tukey’s) (Tables 4–6) showed that Human Osteoblast (HOB) cells grown in the composite’s eluents of both non-porous and porous shows similar results with few difference compared to the negative control (cell media); only the specimens grown at 48 h exposure showed slightly higher number than the negative control, suggesting increasing intracellular activity. However, all specimens cultured up to 72 h exposure had higher viability level compared to the positive control (10% ethanol in media), suggesting that the materials leached by the composites within certain periods (24, 48 and 72 h) are non-cytotoxic (Fig. 14).

**Table 4:** Statistical comparisons of MTT test results of HOB cells exposed for 24 h in eluents of PVA10-CMP without and with gelatin as porogen

24 h exposure	(−) control: media	Composite without porogen	Composite with porogen	(+) control: 10% ethanol in media
(−) control: media		NS	NS	*p* = 0.001 (***)
Composite without porogen			NS	*p* = 0.0003 (***)
Composite with porogen				*p* = 0.0004 (***)
(+) Control: 10% ethanol in media

**Table 5:** Statistical comparisons of MTT test results of HOB cells exposed for 48 h in eluents of PVA10-CMP without and with gelatin as porogen

48 h exposure	(−) control: media	Composite without porogen	Composite with porogen	(+) control: 10% ethanol in media
(−) control: media		*p* = 0.023 (*)	NS	*p* < 0.0001 (****)
Composite without porogen			*p* = 0.0011 (**)	*p* < 0.0001 (****)
Composite with porogen				*p* < 0.0001 (****)
(+) control: 10% ethanol in media

**Table 6:** Statistical comparisons of MTT test results of HOB cells exposed for 72 h in eluents of PVA10-CMP without and with gelatin as porogen

72 h exposure	(−) control: media	Composite without porogen	Composite with porogen	(+) control: 10% ethanol in media
(−) control: media		NS	NS	*p* = 0.0046 (**)
Composite without porogen			NS	*p* = 0.0019 (**)
Composite with porogen				*p* = 0.0019 (**)
(+) control: 10% ethanol in media

**Fig. 15:**
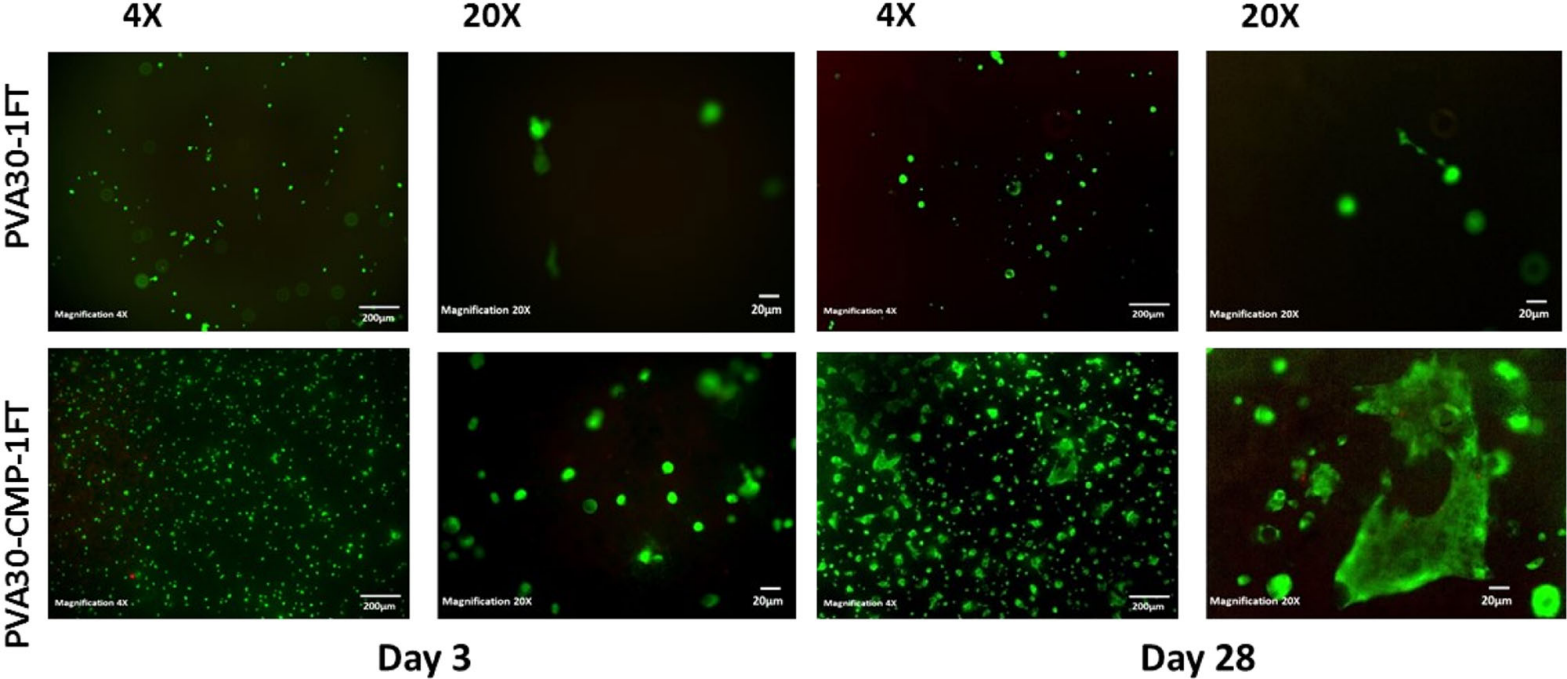
Live/Dead Staining of the HOB cells cultured on the hydrogel and composite for up to 28 days. Fig. 15 showed cell attachment and proliferation on all the scaffolds

A comparison of the live/dead staining of the HOB cells cultured on the hydrogel PVA30-1FT and the PVA30-CMP composite for up to 28 days is shown in Fig. 15. Cell attachment and proliferation on all the composite scaffolds were observed however difficulties were encountered when imaging cells on the hydrogels itself as some cells penetrated and migrated into the internal structure of the hydrogel and fewer cells were visible over time but there was no evidence of extensive dead cells. Osteoblast like cells appeared to have fully spread with a flattened morphology, with visible large extensions and a raised nucleus were observed on PVA30-CMP hydrogel composites. On the PVA30-CMP composites, cells were observed to have spread on the entire surface of the composite with no orientated direction, cells also appeared to be three dimensional and connected to each other, bridging micropores around them forming a three dimensional web. The surface of the composite seemed to be undergoing some early degradation, as granular particles that were assumed to be CMP were observed, exposed on the surface of the composite. It has been reported that on a cellular level, it is likely that roughened surfaces promote the differentiation of osteoblasts and allows the formation a three-dimensional cellular network, which explains the 3D appearance of the HOB cells.

**Fig. 16:**
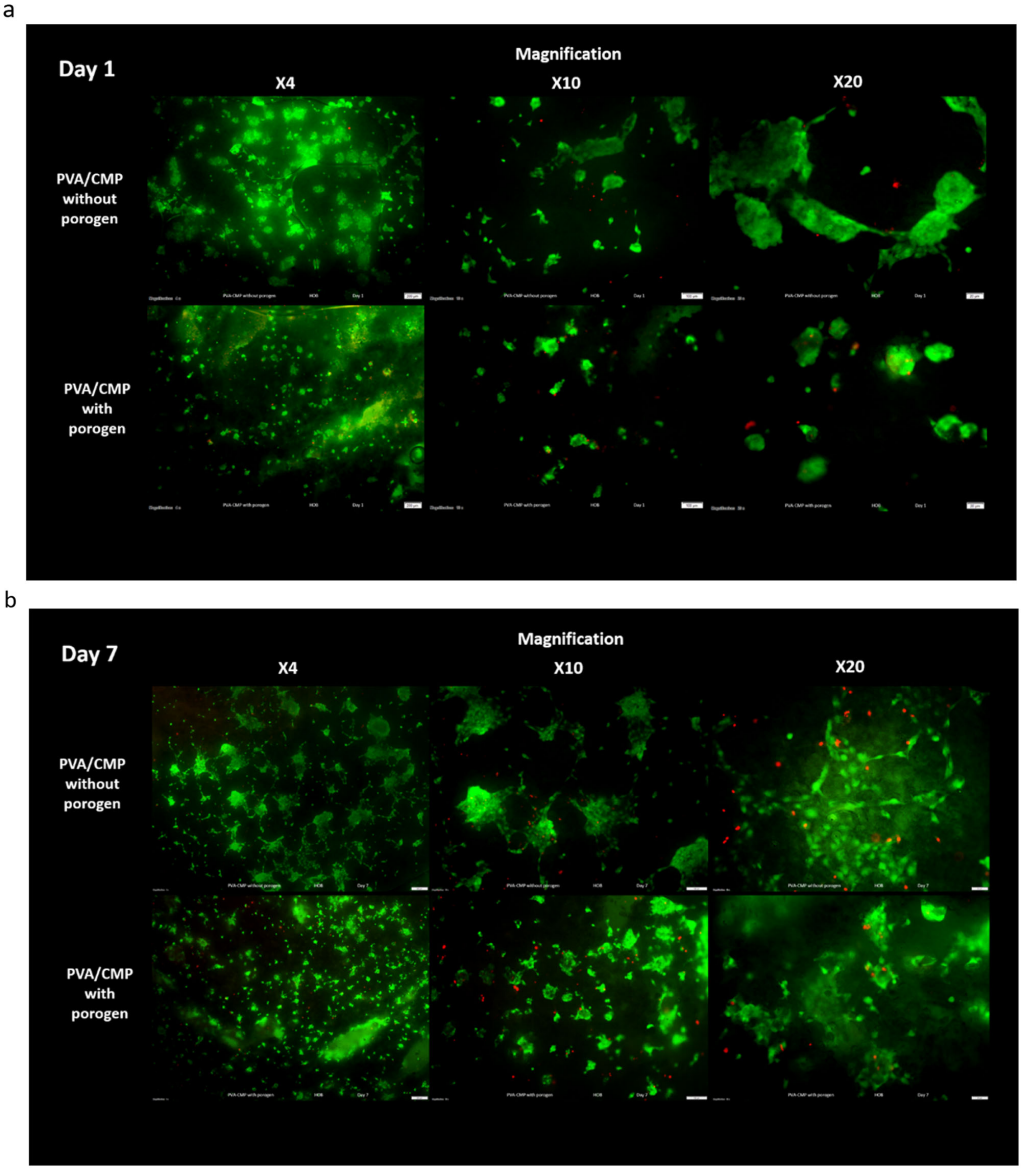

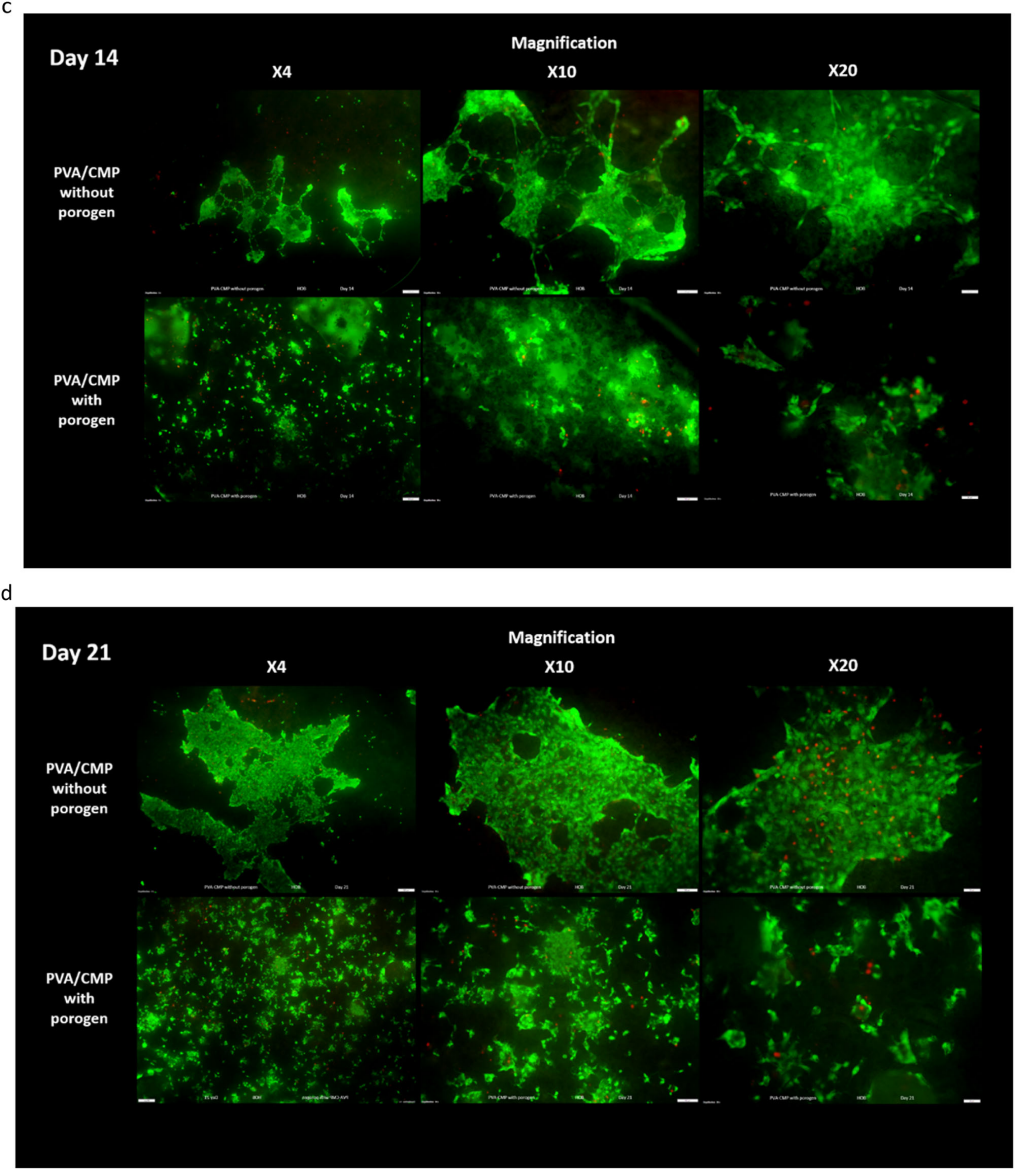
**a** Live/Dead Staining of HOB cells cultured on the PVA10-CMP composites with and without gelatin at days 1 with cell attachment and proliferation on the scaffolds. **b** Live/Dead Staining of HOB cells cultured on the PVA10-CMP composites with and without gelatin at days 7 with cell attachment and proliferation on the scaffolds. **c** Live/Dead Staining of HOB cells cultured on the PVA10-CMP composites with and without gelatin at days 14 with cell attachment and proliferation on the scaffolds. **d** Live/Dead Staining of HOB cells cultured on the PVA10-CMP composites with and without gelatin at days 21 with cell attachment and proliferation on the scaffolds

Live dead staining images of HOB cells on the PVA10-CMP composites with and without porogen are shown in Fig. 16 that clearly indicate that the cells are able to attach on both type of scaffolds and proliferate.

**Fig. 17:**
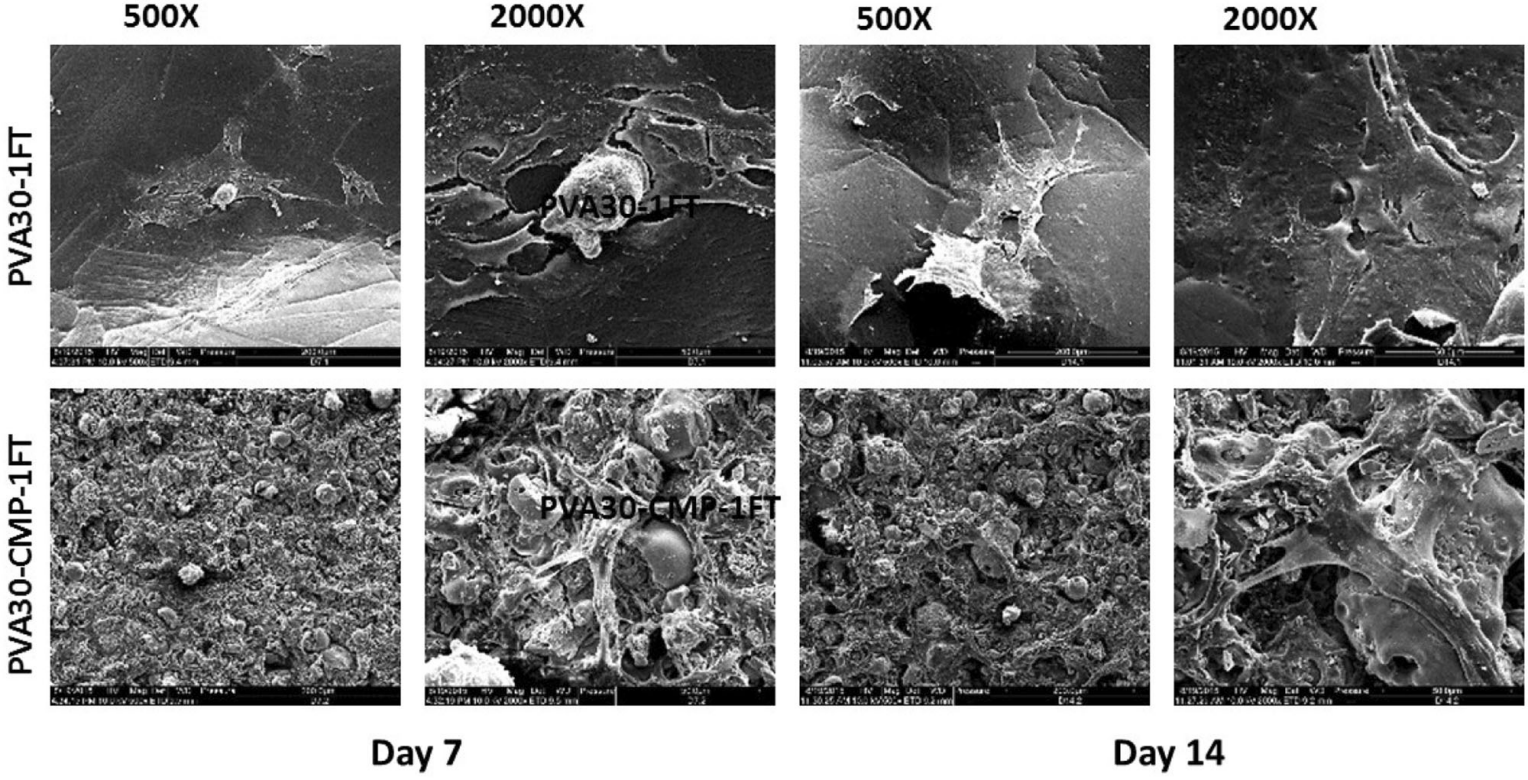
SEM micrographs showing HOB cell attachment on PVA30 hydrogel and PVA30-CMP composite after 7 and 14 days in culture

HOB cell morphology was visualised on the hydrogels and composites on scanning electron micropgraphs after 7 and 14 days in culture (Fig. 17). Cell attachment was observed on both the hydrogel and composite, results displayed in Fig. 17. On the PVA30 hydrogel, the osteoblast cells appeared to have fully spread with a flattened morphology, with visible large extensions and a raised nucleus. After 14 days in culture, very thin capillary-like filopodia extensions were observed and the cells appeared to have been scattered on the surface with no regular orientation. However on the PVA30-CMP composites, cells were observed to have spread all over the surface of the composite, cells also appeared to be three dimensional and connected to each other, bridging micropores around them forming a three dimensional web. The surface of the composite seemed to be undergoing some early degradation, as granular particles that were assumed to be CMP were observed, exposed on the surface of the composite.

## Discussion

PVA is a biocompatible water-soluble synthetic polymer in which the backbone chains are highly interconnected via hydrogen bonding because of the presence of abundant hydroxyl groups. It is a bioinert material hence has been used as a templating agent for precipitation of hydroxyapatite to yield biomimetic composites [11] and functionalised to improve applicability in bone tissue applications. The presence of the pendant hydroxyl groups enables the formation of PVA hydrogels by freeze-thawing process eliminating the use of crosslinking agents and toxic chemicals and additionally the method confers porosity to the gels. The soft elastic nature of PVA and the established biocompatibility of PVA makes it a natural choice as a matrix for formulation of composites. Although a large number of different phases of calcium phosphates have been used as bone grafts and scaffolds for bone tissue engineering, our previous study on a porous calcium metaphosphate scaffold provided extremely convincing results in terms of bone formation and resorbability of the scaffold [9], however the brittle nature limits its clinical handling and yields poor mechanical properties. Thus composite formulation with PVA via bulk blending with the CMP was attempted using different concentrations of PVA whilst elimination of excess PVA prior to freeze-thawing was carried out to yield a ceramic rich composite structure. No coupling agents were used to surface treat the filler particles especially as the CMP is introduced in an aqueous solution of PVA, which when mixed forms a homogenous paste due to the hydrophilicity of the CMP that enables good wetting of the mineral phase. The approach is to allow the gradual resorption of the meta stable calcium phosphate to provide the osteogenic calcium ions, hence it was important not to surface treat the filler. The step of freeze-thawing causes the hydrogel composite to form via crosslinking of PVA, however CMP is also hydrophilic and binds well to the PVA matrix, which is evident from the SEM images shown in Figs. 12 and 13. A set of elastomeric composites were obtained that had rigid sponge like properties yet allowed it to be shaped with a scalpel blade to enable surgical manipulation. The fabrication technique was designed to include the minimal amount of the matrix phase in this case PVA, to enable high concentration of the resorbable ceramic and yet decrease the brittleness of the bone plug. The molecular weight and degree of hydrolysis of PVA were not varied in this study but only the concentration was varied to fabricate the composites via the freeze-thawing approach. The increasing concentration of PVA led to more organised structures thus impacting the physical properties, which is in agreement with previous findings [13]. Furthermore to imbibe higher porosity to the bone plugs, gelatin in its powder form was incorporated and equilibrating the composite at 37 °C in distilled water enabled the swelling of gelatin and soaking in water at temperatures between 37–40 °C allowed a washout to yield macropores as shown in Fig. 13. The FTIR spectra of the composites clearly indicated the presence of both components in the composite (Fig. 4) and there is evidence of weak interaction between the PVA and the CMP specifically due to the peak arising at 1087 cm^−1^ that is attributed to the C-OH bending vibration. The spectral comparison in Fig. 4 of the gelatin imbibed porous PVA-CMP composites shows evidence of the presence of gelatin, which may be attributed to the weak interaction of PVA and gelatin in water [14].

The interaction of fluids with biomaterials is beneficial in understanding the in vivo swelling and fluid uptake, which is essential for designing of biomaterials. Three biologically relevant environments distilled water (DW), simulated body fluid (SBF) and 100% humidity were selected as the interacting environment. The lower concentration of PVA (PVA 10-CMP) in the composites yielded the highest EWC values due to the lowest abundance of the hydroxyl groups and therefore the crosslinking achieved on freeze-thawing. PVA10-CMP had significantly (*P* ≤ 0.032) the highest EWC (29.4, 34.3 and 26.6%) as compared to PVA20-CMP (28.3, 30.5 and 25.1%) and PVA30-CMP (27.0, 21.4 and 21.7%) respectively for SBF, DW and humidity, with the exception of PVA20-CMP in SBF. This observation was due to the dense nature (increase in viscosity) of PVA formed at higher concentrations [15], which results in hindered mobility of the polymer chains, when expanding during hydration, resulting in a limit to the amount of fluids that can be absorbed by the hydrogel network. Under humid conditions the composites are exposed to 100% humidity, however there is no direct diffusion via the surface initially, resulting in reduced overall amount of water molecules absorbed, hence significantly lower values are observed. A shift in the hydration of the hydrogel with changes in osmolarity of hydrating solution can be observed with EWC in distilled water being higher than in SBF. This is due to the fact that SBF contains an ion concentration similar to that of human plasma, while DW in essence has been purified of ionic salts and molecules, therefore osmolarity of the medium in which the hydrogel is hydrated can affect the overall EWC of the hydrogel in a transient manner. A higher salt concentration of Na^+^ in SBF results in more Na^+^ ions being available to bind with the gel, thereby resulting in increased osmotic pressure which results in deswelling of the hydrogel. The bone plugs in this study were designed with the aim of exhibiting lower swelling ratios since excessive fluid uptake can lead to excessive swelling, thus compromising the bone plug, which in turn would exert pressure on the wound defect edges resulting in cell death and necrosis of the surrounding tissue. However a small amount of swelling assists in fitting of the bone plug within the wound without exerting excessive much pressure on the edges. Thus the ability to control the swelling ratio is an important consideration, which can be achieved with these PVA-CMP composites.

Biomechanical properties help to guide surgeons in the use of bone plugs. TruFit CB plugs (Smith and Nephew) are resorbable materials composed of polylactide-co-glycolide (PLGA) copolymer, calcium-sulfate, polyglycolide (PGA) fibres and surfactants. The maximum compressive stresses at failure for the dual layer implants occurred at 5.5 MPa (7 mm), 5.8 MPa (9 mm) and at 8.5 MPa (11 mm) plugs with different diameters. The modulus of elasticity was reported to be 50 MPa (7 mm), 60 MPa (9 mm) and 80 MPa (11 mm), which indicated that the larger the plug size, the higher the strength under test conditions at all strain rates [16], which were in keeping with previous estimated values for successful regeneration of cartilage within a synthetic scaffold. The compressive strength values of the PVA-CMP composites were higher than those of the commercially available TruFit CB plugs (Smith and Nephew) by a factor of over 5 (comparison of PVA30-CMP vs TruFit 11 mm) indicating that the PVA-CMP bone plug composites were mechanically suited. Other comparisons with PVA composites (non-commercial) and calcium phosphate [11] include studies on the compressive mechanical properties of nanohydroxyapatite reinforced PVA gel composite with a compressive strength of 2.91 MPa after 5 cycles of freeze thawing [17] BCP/PVA scaffolds at 0.26 MPa with a PVA concentration of 30 wt% [18] and a PVA/HA composite (15% PVA and 6%HA), which were all significantly lower than those obtained in this study. More recently bilayered hydroxyapatite-poly(vinyl alcohol) composite hydrogels were reported [19] using a combination of directional freezing-thawing and electrophoresis. The compressive were reported to exhibit a gradient mechanical strength depending on the distance to the cathode. The gradient initial tensile modulus were reported from 0.18 MPa to 0.27 MPa and the gradient initial compressive modulus ranged from 0.33 MPa to 0.51 MPa [19].

The decrease in compressive strength after immersion in SBF indicated that the composites undergo some level of polymer degradation and/or CMP dissolution resulting in the weakening of the interaction link between the polymer and ceramic phase, which can be attributed to the interaction of the composite with ions in SBF. This notion was reinforced by further reduction in strength and stiffness of the composites after 4 weeks immersion in SBF. A potential approach in understanding the mechanism of degradation or resorption in vivo is can be achieved by investigating the materials solubility in vitro in physiological fluids [19]. According to Bohner et al [20], “the material should not be soluble in physiological fluids at pH 7.4, because spontaneous rather than osteoclastic dissolution would occur, but should be soluble at a slightly lower pH value, typically between the pH value present at the osteoclast interface (pH 4–5) and pH 7.4”. The experimental composites in this study follow this principle and there is no spontaneous dissolution of the matrix or filler and the rate of resorption of the filler is lowered by the polymer phase, which also acts as a binder and protective barrier to the CMP particles. The reduction in strength and stiffness also indicates that there is no deposition of minerals on the composites during immersion in SBF, as would occur in the case of bioactive glasses such as Bioglass 45S5, this notion is in agreement with results obtained from a study on surface modification of CMP fibres, where they found that no products were formed on the surface of CMP fibres that were soaked in SBF for 30 days at 37 °C [12].

Ideally, a bone graft should possess properties similar to natural bone and have clinically relevant structure to remodelling and regeneration of the native tissue. CMP is a novel scaffold material for tissue engineering and has been demonstrated to possess excellent biocompatibility, osteoconductivity, osteoinductivity and degradability in vitro and in vivo [9] features of which are not detrimental to the process of new bone formation. Biocompatibility and functionality tests were carried out to enable an understanding of how osteoblasts cells would behave in vitro on the formed PVA-CMP composites.

A large volume of cells was observed on the PVA-CMP composites from day 3. Cells were observed to have lodged within the micropores of the scaffolds. From 7 to 28 days in culture, the HOB cells were observed to grow and proliferated within the pores of the composites, this growth and expansion within the pores is observed to become interconnected with cells in the surrounding pores at 21 days in culture, and finally forming what resembles a sheet layer of cells on the outer surface of the composite (this is clearly observed after 28 days in culture). These observations indicate that the PVA-CMP composite is not only biocompatible, but allows for excellent cell proliferation and migration on the composite with minimal cell death they can be accounted to the micropores and lack of interconnected porosity.

## Conclusion

In summary, we successfully fabricated scaffolds by using the resorbable calcium meta phosphate as filler distributed well in a poly(vinyl alcohol) matrix. The CMP particles showed weak interaction with the PVA and significantly improved the mechanical properties of composite scaffolds. With the increase PVA concentration both modulus and strength were found to increase, probably resulting from the increase of the dense elastic network. The scaffolds also showed low degree of swelling and were compressible rendering them suitable as bone plugs. The composites demonstrated mechanical properties suitable for bone tissue engineering applications in medium to low load bearing bone defects. The properties of the composites can also be varied to meet desired needs by simply changing the concentration of polymer content used in fabrication. The incorporation of gelatin to imbibe increased levels of porosity yielded scaffolds without detriment to the mechanical properties. The cell responses with osteoblast like cells suggested that cells could adhere, spread, and proliferate very well in the composite scaffolds, making them promising artificial bone grafts. The efficacy of these scaffolds by virtue of the hydrogel matrix can be further improved by entrapping biological entities such as growth factors either exogenously or endogenously.
